# Enhancing the quality of systematic reviews and meta-analyses

**DOI:** 10.1192/bjo.2025.10876

**Published:** 2025-11-04

**Authors:** Rebecca Strawbridge, Deepika Sharma, Steve Kisely, Ioana A. Cristea, Allan H. Young, Kenneth R. Kaufman

**Affiliations:** Department of Psychological Medicine, Institute of Psychiatry, Psychology & Neuroscience, King’s College London, London, UK; Old Age Psychiatry, Northamptonshire Healthcare NHS Foundation Trust, Kettering, UK; School of Medicine, University of Queensland, Woolloongabba, Australia; Department of General Psychology, University of Padua, Padua, Italy; Department of Brain Sciences, Head of Division of Psychiatry, Imperial College London, London, UK; Departments of Psychiatry, Neurology and Anesthesiology, Rutgers Robert Wood Johnson Medical School, New Brunswick, NJ, USA; Department of Psychiatry, University of Oxford, Oxford, UK

**Keywords:** Systematic review, meta-analysis, evidence, bias, guideline, quality, methodological rigour

## Abstract

Systematic reviews and meta-analyses are often considered the highest level in evidence hierarchies, and therefore are often drawn upon when considering changes in policy. Despite journals implementing measures aiming to enhance the quality of systematic reviews they publish, the authorship raise concerns about the quality of existing and ongoing systematic reviews, particularly relating to transparency and bias minimisation. Building on the current guidelines, standards and tools, we suggest a ‘meta checklist’ which aims to maximise methodologically sound, unbiased and reproducible reviews of the best scientific quality while considering feasibility throughout the process.

Systematic reviews and meta-analyses are often regarded as the highest tier in the hierarchy of evidence, as they synthesise findings across multiple primary studies using rigorous and standardised methodology. In principle, this results in more reliable answers to clinical and research questions than an individual study can provide.^1^ They are impactful in developing and updating treatment guidelines,^2^ which can, and arguably should, lead to improvements in the quality of care delivered in everyday clinical settings.^3^ Consequently, the impact of systematic reviews and meta-analyses extends beyond clinical practice, influencing healthcare policy and economics, while also serving as critical foundations for shaping future research priorities.

However, the value of a systematic review, with or without an accompanying meta-analysis, is contingent upon its methodological rigour, transparency and minimisation of bias.^4^ Given the potential harms that biased systematic reviews could have, numerous handbooks, associations and guidelines are available to ensure methodological robustness and rigour. These resources are referenced and utilised throughout this article.

In the last decade or so, academic journals have increasingly implemented measures to improve the quality of the systematic reviews that they publish. These include mandating adherence to the PRISMA (Preferred Reporting Items for Systematic Reviews and Meta-Analyses) guidelines, typically through the submission of the PRISMA’s checklist and flow diagram,^5^ as well as encouraging or requiring the pre-registration of review protocols (e.g. via the International Register of Systematic Reviews (PROSPERO)).^6^

Despite these efforts, such requirements can at times become procedural formalities, leading to what could be described as a ‘tick-box’ approach. While some evidence suggests that PRISMA endorsement^7^ or citation^8^ is associated with improving reporting completeness, numerous studies have demonstrating that even systematic reviews citing PRISMA frequently fall short of reporting across all checklist items.^8–10^

As editors, we regularly encounter methodological and reporting concerns when peer reviewing systematic reviews, and such issues are also evident across published literature. Others have published articles purporting a potential decreased value of some systematic reviews, citing concerns related to redundancy, low methodological quality and susceptibility to bias. These shortcomings may result in conflicting, low-utility or misleading conclusions, which undermine the objective of informing evidence-based practice. Concerns raised include topic duplication, selective reporting, industry influence and limited clinical applicability.^4,11^ Collectively, these observations highlight the importance of not only critically assessing whether a systematic review is justified for a given research question but also ensuring the conduct of such reviews adheres to high standards of quality, transparency and relevance.

### Aims

This article aims to serve as a practical and evidence-informed resource for authors, peer reviewers, journal editors and readers of systematic reviews. For authors, we recommend its consideration throughout the entire review process, from initial planning and protocol development to manuscript submission and revision. Specifically, we first summarise: (a) key existing frameworks designed to optimise the conduct and reporting of systematic reviews; (b) established tools and criteria for evaluating the quality of systematic reviews and (c) common drawbacks observed in current systematic reviews, along with the underlying causes of these issues. Building on this foundation, we integrate insights from existing guidance, tools and standards to propose a set of *pragmatic*, consensus-informed criteria for systematic reviews for conducting and appraising them. These criteria are intended to ensure that systematic reviews meet a threshold of methodological rigour, transparency and relevance appropriate for publication in reputable journals, including *BJPsych Open.* Importantly, this proposed framework is not intended to replace existing guidelines, but rather to complement and signpost to them, offering a coherent and practical tool to support best practice at every stage of the review process.

## Methods for developing our systematic review criteria

A systematic review is a type of study design (see also^12^) that provides a clear research question and details the sources searched, including the databases and search engines used, the search date and the complete search strategy. It specifies the criteria for including or excluding studies, describes the methods for screening and selecting studies, assesses and reports the risk of bias (RoB) of the included studies, and provides information on data analysis and synthesis to ensure the results can be reproduced.

Several established, consensus-based frameworks are available to support the rigorous conduct of systematic reviews, and to minimise bias. Among these, guidance developed by the Cochrane Collaboration and PRISMA are particularly foundational. Our proposed criteria were developed through a multi-step process: (a) review of current methodological guidelines (e.g. the Cochrane Handbook, PRISMA); (b) consulting existing tools for assessing systematic review quality and RoB; (c) critical reflection on the authors’ collective experience of both conducting and peer reviewing systematic reviews, including common drawbacks and their underlying causes. Final agreement on the proposed criteria was achieved through iterative written feedback among the authorship team, followed by a consensus meeting.

We note that this article focuses on generating high-quality systematic reviews with consideration of the RoB both within studies included in the review, and consideration of the overall certainty of evidence reviewed. Since the term quality is widely used with different specific meanings, including overlap with other terms, we state the definitions of respective terms used in this article in Table 1.

**Table 1:** Definitions of quality, risk of bias (RoB) and certainty as used in the current article

Term	Definition in this article	Alternative possible definitions or uses	Limited examples of tools used to assess construct
Quality	The extent to which a systematic review is conducted according to best practice.	Sometimes used interchangeably with RoB or certainty (as per our definitions).	Risk of Bias in Systematic Reviews, Measurement Tool to Assess Systematic Reviews
RoB	Specifically the potential for systematic errors in *individual* included studies’ design, conduct or reporting that may distort the true effect of an intervention.	Sometimes used specifically in reference to the Cochrane RoB tool for examining randomised controlled trials.	Cochrane RoB tool, Risk of Bias in Non-Randomised Studies
Certainty	An assessment of the overall body of evidence reviewed is important to determine the level of confidence with which we can trust that the reported effects reflect a true effect across studies.	Used less often than the other terms defined here.	Grading of Recommendations Assessment, Development and Evaluation

### Consulting current guidelines

The Cochrane Collaboration is a multidisciplinary, globally recognised organisation that produces collaborative systematic reviews and offers a range of resources to support their development. These include an in-depth methodological handbook, training modules and dedicated software for meta-analysis. The *Cochrane Handbook for Systematic Reviews of Interventions* provides detailed guidance on core systematic review methodology, with a particular emphasis on the appraisal and synthesis of (RCTs).^13^ This is supplemented by an online course covering the entire systematic review process through 11 structured modules and an assessment.^14^ Additional online courses on the fundamentals of systematic review and meta-analyses methodology are available through institutions such as John Hopkins university^15^ and the Centre for Reviews and Dissemination.^6^

While many of the resources focus primarily on intervention reviews, guidance is also available for conducting systematic reviews and meta-analyses from a variety of study designs and research aims (e.g. prognostic factors, diagnostic accuracy, experiential/qualitative, economics, prevalence, risk, policy, psychometrics and methodology). Resources also address how to appropriately select the type of review and formulate robust research questions.^16^

Authors are strongly encouraged to adhere to the PRISMA guidelines (originally published in 2009,^17^ and most recently updated in 2020^5^). PRISMA provides a comprehensive checklist and flow diagram designed to enhance the transparency and completeness of reporting. Several PRISMA extensions exist for specific review types including: PRISMA-P for protocols,^18^ PRISMA-ScR for scoping reviews,^19^ PRISMA-DTA for diagnostic test accuracy,^20^ PRISMA-IPD for individual patient data analyses^21^ and PRISMA-NMA for network meta-analyses.^22^

To assist with the appraisal of primary studies included in systematic reviews, a variety of RoB tools are available, tailored to specific study designs. For RCTs, the *Cochrane RoB 2* tool is widely recommended.^23^ The Cochrane Handbook also offers guidance on assessing RoB in randomised^24^ and non-randomised^25^ interventional studies as well as diagnostic accuracy reviews.^26^ In addition, others have developed appraisal tools suited to different methodologies.^27^ Notably, the Joanna Briggs Institute provides structured RoB tools for a broad range of study types.^28^ After synthesising the findings, the Grading of Recommendations, Assessment, Development and Evaluation (GRADE) approach provides a structured method for assessing the overall certainty of a body of evidence within a systematic review. It is widely used to support the development of clinical practice guidelines and evidence-based recommendations.^29^

A number of additional resources focused on maintaining quality standards throughout the systematic review process were also reviewed in the preparation of this article, e.g.^30^

### Consulting existing measures to ensure review standards

Authors should follow guidelines to ensure the quality of their review. While most resources cited focus on intervention/efficacy systematic reviews, we aim to signpost readers to best-practice resources and guidelines across systematic review types, non-denominationally. Indeed, many principles and criteria pertain across systematic review types. Since the 1980s the quality of systematic reviews has been considered on sets of criteria,^31,32^ closely followed by early validated tools intended to rate the methodological quality of interventional systematic reviews (e.g. Overview Quality Assessment Questionnaire (OQAQ))^33^. Others took a clinically focused approach to evaluate the quality of intervention or prognostic systematic reviews.^34^ A MeaSurement Tool to Assess systematic Reviews (AMSTAR) was developed and validated to address missing criteria from the OQAQ,^35^ subsequently revised (R-AMSTAR) to include a quantitative scoring method to assess quality^36^ and later as ‘AMSTAR-2’ which incorporated non-randomised as well as randomised interventional reviews.^37^ AMSTAR-2 remains widely used and criteria from this (as well as PRISMA and others) were included in the subsequent Methodological Expectations of Cochrane Intervention Reviews (MECIR) guidelines to specify method and reporting standards for Cochrane intervention protocols/reviews.^38^ The Risk of Bias In Systematic Reviews (ROBIS) tool was developed and validated to examine RoB in systematic reviews more broadly (i.e. not just interventional).^39^ Recently, the Critical Appraisal Tool for Health Promotion and Prevention Reviews (CAT HPPR) was developed, through first identifying existing appraisal tools (amounting to 14), and then consensus-developed quality criteria, rating system, expert input and finalisation.^40^ The CAT HPPR attempts to be applicable to various reviews, including specific criteria for rapid and scoping reviews in addition to systematic reviews. Below, we note consideration of our key guideline points in relation particularly to CAT HPPR. We highlight that these tools are primarily used for retrospectively determining the quality of systematic reviews rather than as tools to enhance quality prospectively or concurrently, as our criteria attempt to.

### Common issues identified in systematic reviews by the authors

We note commonly encountered drawbacks of reviews submitted to this and other journals during editorial and peer review considerations. These are discussed summarily below.

The gold standard for systematic reviews includes dual and independent screening for titles and abstracts, dual and independent full-text reviews, dual and independent extraction, dual and independent/RoB assessment and examination of the certainty of the synthesised evidence.

We frequently observe single (as opposed to dual) reviewers undertaking these steps, often without justification for doing so. In some cases, a (usually small, e.g. 10%) proportion of articles are screened/reviewed by a second independent reviewer, with or without a statistic denoting inter-rater reliability. Sometimes, data are extracted by one individual, with a second reviewer checking the single-reviewer’s extracted data (or a portion of it) for accuracy. As described further below, these examples stray from best practice and increase the RoB of a systematic review. Another common issue is extended periods of time between a review’s last literature search update and manuscript submission/publication, which renders a review essentially outdated.^41^ It is also not unusual to read manuscripts that claim to adhere to PRISMA guidelines, but in fact omit information required by the PRISMA checklist (e.g. statement of which reviewer(s) undertook which roles or missing search terms.) Finally, there are often discrepancies which can be found between a (cited) *a priori* protocol and a final manuscript which are not mentioned or justified.

There may be several explanations for why these methodological issues occur. Our experience indicates that often these projects are unfunded and there is an expectation that they can be completed as straightforward, low-resource studies. Consequentially, a review team is not able to dedicate sufficient specialist expertise who can provide supervision to ensure that a) sufficient resources are allocated to undertake the systematic review according to best practice, b) sufficient scoping is undertaken to develop a protocol which is comprehensive and realistic and that c) complex activities (e.g. analyses) are completed with due rigour. Often, as a result, scoping does not adequately inform the resources required, which can lead to compromised quality (such as single reviewer activities and inability to update searches in time for timely publication, as above), and lack of expertise in designing search strategies and conducting analyses further that increase the RoB.

We discuss guidelines in the following sections.

### Systematic review steps

Box 1 displays the required aspects of reviews, which are expanded upon below.

Box 1 Steps to undertake a systematic review and meta-analysis (adapted from Kisely & Siskind)^42^

**Table:**
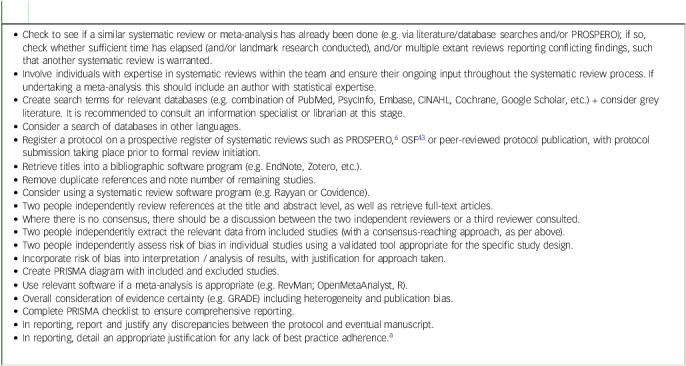


Check to see if a similar systematic review or meta-analysis has already been done (e.g. via literature/database searches and/or PROSPERO); if so, check whether sufficient time has elapsed (and/or landmark research conducted), and/or multiple extant reviews reporting conflicting findings, such that another systematic review is warranted. Involve individuals with expertise in systematic reviews within the team and ensure their ongoing input throughout the systematic review process. If undertaking a meta-analysis this should include an author with statistical expertise. Create search terms for relevant databases (e.g. combination of PubMed, PsycInfo, Embase, CINAHL, Cochrane, Google Scholar, etc.) + consider grey literature. It is recommended to consult an information specialist or librarian at this stage. Consider a search of databases in other languages. Register a protocol on a prospective register of systematic reviews such as PROSPERO,^6^ OSF^43^ or peer-reviewed protocol publication, with protocol submission taking place prior to formal review initiation. Retrieve titles into a bibliographic software program (e.g. EndNote, Zotero, etc.). Remove duplicate references and note number of remaining studies. Consider using a systematic review software program (e.g. Rayyan or Covidence). Two people independently review references at the title and abstract level, as well as retrieve full-text articles. Where there is no consensus, there should be a discussion between the two independent reviewers or a third reviewer consulted. Two people independently extract the relevant data from included studies (with a consensus-reaching approach, as per above). Two people independently assess risk of bias in individual studies using a validated tool appropriate for the specific study design. Incorporate risk of bias into interpretation / analysis of results, with justification for approach taken. Create PRISMA diagram with included and excluded studies. Use relevant software if a meta-analysis is appropriate (e.g. RevMan; OpenMetaAnalyst, R). Overall consideration of evidence certainty (e.g. GRADE) including heterogeneity and publication bias. Complete PRISMA checklist to ensure comprehensive reporting. In reporting, report and justify any discrepancies between the protocol and eventual manuscript. In reporting, detail an appropriate justification for any lack of best practice adherence.^a^

PROSPERO, International Register of Systematic Reviews; OSF, Open Science Framework; Embase, Excerpta Medica Database; CINAHL, Cumulative Index to Nursing and Allied Health Literature; RevMan, Review Manager; GRADE, Grading of RecommendationsAssessment, Development andEvaluation; PRISMA, Preferred Reporting Items for Systematic Reviews and Meta-Analyses.

a. In general, limited resources, where this could have been anticipated and planned ahead for, would not be considered an appropriate justification for lack of best practice adherence. However, there may be circumstances in which this is justifiable, and the authors note that most guidelines are focused on intervention efficacy or other areas with concerning consequences if compromised quality exist that may be lower in other systematic review topics.

### Protocol preparation

This is important for formulating a valid, considered research question and requires sufficient scoping to determine that a question has not already been answered, there is an appropriate review methodology (which may not always be a systematic review; below) and that there appears to be sufficient evidence from primary studies. The scope and methods used in systematic reviews must strike a balance between being feasible and being comprehensive, as well as being appropriate otherwise in design.^16^ Scoping here critically involves a search of current protocols to verify that another overlapping review is not likely being conducted. In support, we cite here an incidence of several overlapping reviews being published in close time proximity (several of which contained errors)^44^ and examples of ‘empty’ reviews, where no eligible studies were identified.^45^ This stage includes establishing whether a systematic review is the optimal methodology as opposed to say a scoping review^43^ or rapid review^46,47^ or a ‘robust critique’ method.^47^ There may be instances where, despite sufficient scoping and registration of a protocol (below), an alternate approach is subsequently realised as optimal, in which case the amended protocol should still be cited, along with a justification for the change. It is important from the outset to involve people in the review team with expertise in systematic review methodology to ensure that the review team have had adequate training, that scoping and planning are conducted thoroughly according to best practice and that there is sufficient staffing resource to complete the project. Input from an information retrieval specialist should facilitate construction of a search strategy that is sensitive and specific to the research question, that the search strategy – across databases – detects pre-defined marker papers and is feasible. Input here from an author with statistical expertise in meta-analysis is also needed, if such a synthesis is envisaged. If a meta-analysis is not envisaged, a systematic methodology to synthesis should be consulted, e.g. Synthesis Without Meta-Analysis (SWiM).^48^

All subsequent stages should include regular check-ins with the author who has systematic review expertise to support the reviewers and check protocol adherence as well as make informed team decisions in cases where additions to, or discrepancies from, the protocol are indicated.

### Protocol publication

As well as having an *a priori* systematic review protocol, the importance of registering protocols is clearly critical for transparency and RoB. Examples include PROSPERO,^6^ and the Open Science Framework (OSF)^49^ for reviews not covered by the former. For these reasons, it is important that protocols are registered after scoping but before data extraction. Ideally, they should be registered prior to screening of studies against eligibility criteria, although PROSPERO, for one, permits registration before data extraction is initiated.^6^ Despite many journals now requiring the protocols of systematic reviews to be registered, this is still not universal practice, and we cite supporting evidence here that prospective protocol publication/registration is associated with improved review methodology.^50^

Some journals publish protocols (following peer review), and having a peer-reviewed protocol may help to further improve the methodological quality. However, many journals do not in most circumstances (including the *British Journal of Psychiatry Open*) as platforms such as PROSPERO exist and are structured to ensure that all relevant methodological considerations are covered. Having most systematic review protocols on PROSPERO facilitates ease of scoping for others planning reviews and permits time-tracked updates, including stage-of-review completion.

### Search procedures – where, when, what

*Where:* databases and sources searched should be sufficient to identify all relevant evidence to answer the question asked. This will differ based on the question or field of research. Best practice guidelines recommend searching several databases (Cochrane requires ≥2); in our field this should often include MEDLINE and Embase. Handsearching is also needed, for which further detail is available elsewhere.^51^ Clinical trial registries are increasingly recommended as part of this search.^51^ Recommendations regarding searches of grey literature are contrasting, with some suggesting that a lack of clear peer-reviewed output is problematic for reasons of bias, while others argue that grey literature inclusion addresses issues including those surrounding publication bias.^51^ Good practice in considering grey literature and further reasons for a considered approach here are available.^52,53^ We would recommend articles justify the inclusion or exclusion of grey literature based on these considerations and the focus of the review in question.

*When:* The systematic search should cover the full range of time that the research question is relevant for, which in many cases is the inception of the database until the date of search. Unless there is a clear reason (e.g. smartphone research, for which only the most recent few decades would be relevant), any discrepancy from this would need to be justified. So as to remain a timely systematic review, the time between the search and the final manuscript submission should be as short as possible (although 12 months is often considered a maximum duration between the search and manuscript submission, this is not always appropriate or possible)^41^ and it is recommended to update the literature search prior to manuscript submission. Sometimes, in the event of a long duration after manuscript submission (e.g. multiple rounds of peer review), it may also become appropriate to update searches to ensure a timely systematic review.

*What:* The terms used in a search must be comprehensive enough to include all research relevant to the research question and study eligibility. Terms should consider all of the PICOS (population, intervention, comparisons, outcomes, study design) framework and for each component of their PICOS, use as many relevant terms as possible (which can sometimes include unusual or outdated terms). PICOS is primarily focused on interventional research but is adaptable towards other designs; for observational studies, PECOS (substituting ‘exposure’ for ‘intervention’) is often used. Scoping (number 1, above) is important to ensure that search terms are sufficiently complete to identify all relevant evidence while maintaining feasibility (i.e. not returning so many results that cannot be feasibly reviewed). The search should also maximise inclusivity with regard to language (i.e. including articles not in English if at all practicable). This is due to warnings of increased bias from reviews not including studies not written in English, as significant results are more likely to be published in English language journals.^54^

As with other sections, the full search procedures must be reported in manuscripts. We cite evidence that reporting around search procedures is often poor; one examination of 272 systematic reviews published in 2012 from high-impact paediatrics, surgery or cardiology journals reported that most articles named the search terms used, but only 33% provided all details of the search undertaken and only 22% reported the search date. Only 13% of studies provided reproducible search strategies for all databases searched.^55^ While it would be hoped that this has improved following the common use of the PROSPERO and such pre-registration platforms that require at least some *a priori* search details, a very recent article indicated that only ∼10% of 453 searches had reported sufficiently to ensure that searches could be reproduced within 10% of results, e.g. if an original search reported 1000 articles retrieved, the attempt at reproduction yielded 900–1100). Some original searches’ results differed from the reproduced search by more than 1000% and only one was fully reproducible.^56^

### Review procedures (initial screening, full text review, study inclusion and data extraction)

*Initial screening:* Retrieved articles’ titles and abstracts should be screened by at least two people independently of one another and blind to each other’s ratings (‘dual screening’). Cochrane’s guidance states that for this stage, duplicate screening is ideal, but that ‘it is acceptable that this initial screening of titles and abstracts is undertaken by only one person’ if it is unfeasible to meet this.^51^ An alternative that has been proposed if needed is for only a proportion of articles to be screened by two individuals (with a reported statistic of agreement) and that for that proportion any article selected by one, but not the other, reviewer, would be considered in full. However, even if 50% of 1000 articles were screened by two people with 90% agreement, this could confer a potential 50 articles discarded that may have been eligible and would otherwise have been considered in a full-text screen. We argue therefore that not undertaking dual screening substantially increases the likelihood for excluding eligible evidence.

*Full-text review:* For those studies not excluded at the previous stage, the full articles are considered comprehensively here against the eligibility criteria. As above, Cochrane does state that ‘it is essential, however, that two people working independently are used to make a final determination as to whether each study considered possibly eligible after title/abstract screening meets the eligibility criteria based on the full text of the study report(s)’.^51^

*Study inclusion/resolving discrepancies:* As above, and Cochrane states that a process should be pre-specified for resolving disagreements or discrepancies; this may initially be between the two reviewers and/or with a third reviewer or the whole study team.^51^

*Data extraction:* As above, we echo other guidelines stating that data ’should be extracted independently by at least two people’.^57^ We emphasise that it is generally insufficient for one reviewer to undertake extraction and another to check the completed extraction.

In support of dual procedures by two reviewers independently, it is noted that over 50% (and up to 100%) of reviews contain errors, often related to the ineligibility of included studies^58^ and that errors in data extraction are partially attributable to inconsistent findings between different reviews asking the same question (e.g. ∼20% extraction errors from one comparative investigation).^44^

### RoB assessment

As described above, different RoB tools are appropriate for different types of systematic reviews and original study design, and much guidance around this exists.^24,25^ Some use the terms ‘RoB’ and ‘quality’ assessment interchangeably; however, even for non-interventional studies the term ‘risk of bias’ is more relevant to systematic reviews in that rather than how well a study was conducted, we are interested in how likely or how much the results might be inaccurate as a result of the study methodology. In this article we use the term RoB to denote the risk of bias incurred by individual studies included within a review, certainty to denote the extent to which we believe the overall review findings (see below) and quality to denote how rigorously a systematic review itself was conducted (as above); see also Table 1.

Dual and independent assessment of RoB is required to reduce subjectivity and errors, and authors should transparently report how disagreements were resolved. In reality, discrepancies between raters are extremely common.^59^ Notably, the same primary study is often rated differently between tools^60^ and between reviews.^61^ This highlights that, despite the existence of structured guidance, RoB assessments are inherently subjective to some degree, and this can meaningfully affect review conclusions. This can include if RoB ratings are used in meta-regression or sensitivity analyses. To improve transparency and interpretability, authors should provide an explicit and well-justified rationale for RoB judgements of studies in systematic reviews, which can allow readers to critically assess the reasoning behind these assessments and their agreement with the interpretation of findings. A ‘robust critique’ approach can be utilised to provide transparent, structured narrative assessments of methodological strengths and weaknesses.^61^

While validated RoB tools offer consistency, we suggest that it can be acceptable for validated RoB tools to be modified to suit a specific systematic review question, if adequate justification is provided and, ideally, it is pre-specified in the systematic review protocol.^62^ Disagreement exists as to whether an *overall* RoB score should be attributed to included studies; although summary scores can be an easily interpretable indicator of RoB, these can obscure important domain-level information and falsely imply objectivity.^63^ Stratification into ‘high’ versus ‘low’ RoB may also oversimplify nuanced assessments and introduce further subjectivity. PRISMA (2020) outlines the importance of implementing RoB assessments, first in the RoB of individual studies and second during the review synthesis stage. Within data synthesis, or analysis, reviewers must consciously ensure they are not entirely discounting the findings of studies that are methodologically less rigorous, while also not treating all studies equally. If analyses are desired including only studies judged to be at low RoB, it is good practice to perform a sensitivity analysis to compare how results would differ if high or uncertain RoB studies were included. Another approach is where analyses are stratified according to the overall RoB, providing multiple estimates to observe the potential for results to change when removing less robust studies.^60^ However, subjectivity in RoB assessments must be considered carefully in analytic weightings, as noted above.

### Judging the certainty of the evidence

An assessment of the overall body of evidence reviewed is important to determine the level of confidence with which we can trust that the reported effects reflect a true effect across studies. We refer to this as a ‘certainty’ assessment, although the term ‘quality’ is sometimes used in this context (see Table 1). The most widely used approach is GRADE (as above), which guides reviewers in rating the overall certainty of evidence for each outcome.^64^ GRADE evaluates five domains spanning RoB, inconsistency, indirectness, imprecision and publication bias. The robustness of designs included in the systematic review is incorporated (high for RCTs and low for observational studies) and ratings can be altered up or down based on the five examined domains, to ultimately assign a certainty rating of high, moderate, low or very low. This process, while structured, involves subjective judgements that can meaningfully influence how findings are interpreted and the resultant recommendations made.^65^ As with RoB, systematic review authors should provide an explicit and well-justified rationale for certainty assessments. If reviewers consider a certainty judgement inappropriate, a strong justification should be given, since without this, readers cannot fully evaluate the robustness of the conclusions.

### Analysis

Before deciding to undertake a meta-analysis, reviewers must make an evidence-based decision on whether a statistical analysis will yield accurate results. If in doubt as to the appropriateness of a meta-analysis, it could be preferable to choose a narrative synthesis, rather than report an effect size that may be uninformative or, worse, misleading.^66^ There are guidelines for narrative syntheses^48^ in addition to the widespread recommendations for conducting meta-analyses, both universally^67^ and specific to for example interventional^66^ or observational/epidemiological^68^ research.

If not undertaking meta-analysis, it remains critical to describe (ideally in an *a priori* protocol) the narrative methods used to synthesise the evidence reviewed. A SWiM (or similarly methodical) approach is considered appropriate.^48^

If undertaking meta-analysis, it is critical to both measure and explore heterogeneity between the results of individual studies^69^ and there are several methods for doing so.^70^ Heterogeneity is the rule rather than the exception in meta-analyses and can often be anticipated, particularly when multiple populations, interventions, comparisons, outcomes and/or study designs are included (as per PICOS/PECOS). Such sources of heterogeneity are often evident from the outset and should be considered during the planning phase – both when setting eligibility criteria and when deciding whether to conduct a meta-analysis and how to explore heterogeneity. The decision to use a fixed- or random-effects analysis should be made (and stated) a priori based on the expected degree of heterogeneity; unless there is a case for anticipating the true effect to be common and consistent across studies (in which case fixed-effects could be justified), a random-effects model would be supported (based on the expectation that the true effect is heterogeneous between populations). Authors need to consult guidelines^66,71^ and explicitly justify the approach taken.^72^ Again, broadly, it is important to ensure meta-analyses meet assumptions,^66^ and deal appropriately with issues such as missing data, imputation and conversion between units.^66^ This applies also if meta-analyses are unusual in some respect (for one specific example, rare event outcomes^73^). These and other important considerations should be planned for as much as possible a priori, for example when multiple data could be used and need to be selected from (e.g. multiple questionnaires examining the same outcome, or multiple timepoints, models or participant subgroups). These decisions should ideally be pre-specified in a statistical analysis plan to reduce bias and increase transparency. Tools such as the one developed by Higgins et al^74^ can assist in this process. Their structured checklist comprises 43 items across domains such as data sources, analysis methods and interpretation, and helps reviewers systematically assess the planning and conduct of a meta-analysis, including how flexible analytical choices were made and justified. Another important consideration should be the software used for meta-analysis, which differ in several ways and many of which require close attention by reviewers, e.g. default settings and implementation of models, particularly for complex data. This highlights the need for statistical supervision as well as careful software selection.^75^ The analytic code, model(s) and outputs should also be checked by a second reviewer. Potential publication bias is another factor which is important to consider and may necessitate searches of relevant clinical trial registries.^76^ Although issues surrounding publication have likely improved through initiatives such as mandatory publication clauses, there are still examples of clinical trials that are unpublished and/or are published with key findings unreported.^77^

In terms of reporting meta-analyses, we emphasise that, similar to other systematic review aspects, existing articles often do not adhere to best practice. One study examining meta-analyses found that only 54% reported quantitative tests in sufficient detail that they could be recreated, and with some particular omissions in reporting, e.g. access to datasets/code (only mentioned by 30% of the systematic reviews examined).^78^ A variety of other errors in meta-analyses also occur frequently (e.g. in analytic approaches, extraction, manipulation and interpretation.^79^

### Reporting

As alluded to in several of the above sections, and covered by PRISMA checklists, all aspects of systematic reviews need to be reported comprehensively, in such detail that they could be recreated. We highlight below some specific omissions observed frequently.

*Changes to methodology after protocol:* Not everything can be planned and sometimes it is needed to amend aspects of the review. It is important that in a final manuscript these are specified and justified. Although updates to protocols can be made in PROSPERO^6^ or OSF,^49^ it is also important to clearly state in manuscripts these aspects and their justification, as changing methodology after an event may introduce bias. For example, eligibility criteria could be amended to exclude a study or amend outcomes that do not align with authors’ expectations.

*Missing analytic details:* We highlight that reporting of analytic details in meta-analyses is important and often incomplete. One example is being aware of the difference between s.d. and s.e. (or other variance statistics) and the need to convert from one to the other when standardising data for analysis. It is crucial to clearly report the method of conversion used, as incorrect conversions can lead to inflated effect sizes and narrower confidence intervals, which may significantly alter the conclusions of the analysis, for example here^80^ with further detail here.^81^ Reporting approaches to address missing data are similarly important here.

We acknowledge that incomplete reporting (such as non-use of PRISMA) does not in itself reflect a poorly conducted systematic review – rather poor reporting.^82^ Poor conduct may or may not reflect high bias (e.g. a lack of pre-registration may or may not induce bias by altering the methodology or focus after conception), although other elements covered here have direct implications (such as objective errors). One review of quality assessments of systematic reviews, conducted for those published between 1990 and 2014, examined the overall results of these methodological, and/or reporting, quality ratings. Across 56 articles, good adherence for some items (e.g. rationale for review, study eligibility criteria) and poor for others: only 6% reported on protocols, 9% included a flow diagram of search results and inclusion procedures; only 30% had studies assessed for eligibility and the data extracted by two individuals; only 37% assessed RoB (NB these results are of the total comparisons made on each respective specific item).^83^ Similar findings have been reported in other specific fields, including recently (up to 2018).^84,85^ Conversely, enhanced transparency can be delivered via improved reporting (compared to most systematic reviews except Cochrane reviews) by including a list of studies in the supplementary material that were excluded after full-text review.

## Further considerations

This is not a comprehensive guide and others have greater depth of details throughout the process, including those we have not touched on here such as for example reports from multiple studies, contacting original authors where information is missing^51,57^ or finding full texts.^51^ We have not covered the use of automatic tools in any part of the systematic review process, although the development and use of these is increasing. At this time, we are not able to recommend these (given a lack of thorough validation); however, this is to be revisited in light of the emerging and rapid progress in this field. It is first required that such a tool is subject to rigorous validation.

The focus here has been on clinical research and, as often the case, tends to lean towards the effects of interventions on patients’ well-being/health. Many of these points are relevant also to other types of systematic reviews and guidelines exist for different types (including PRISMA extensions for different review types), e.g. pre-clinical questions,^86^ overviews of reviews (also termed meta-reviews or umbrella reviews)^87,88^ or ‘living’ systematic reviews.^89^ We emphasise that other types of systematic literature review require comparable rigour to the guidelines for systematic reviews described in this article.

Although this article is not focused specifically on scoping reviews, we also experienced several scoping reviews that have, for example, only undertaken screening and/or full-text review and/or data extraction by a single reviewer (or only a minimal proportion were undertaken dually). We highlight that the key difference between a systematic and a scoping review is in the type of question(s) asked and the specificity of the outcomes considered, rather than the rigour to guidelines for the systematic review methodology described in this article. A key difference is that RoB is typically not assessed in scoping reviews, but the justification is related to the broadness of the questions considered, including that studies are likely to be more heterogeneous which challenges attempts to compare their quality; additionally,the interpretation of results differs from a systematic review which intends to pool similar studies with similar outcomes. As mentioned, guidelines for scoping reviews are available elsewhere, e.g. reviews (PRISMA-ScR).^19^ As with systematic reviews, pre-registration of a protocol is good practice although is often through the Open Science Framework as PROSPERO does not accept these reviews.

The points covered here and elsewhere evidence the complexity and rigour required in undertaking a systematic review. Consequently, we note that peer review of systematic reviews is an intensive process. With peer review essentially constituting an altruistic citizenship activity, many systematic review peer reviewers are not adequately resourced to input sufficient resources into conducting thorough reviews. This should, in order to do due diligence to the process of detailed protocol registration, include an in-depth comparison between the protocol and the eventual manuscript (although the latter should detail and justify any discrepancies between them). Systematic review peer reviewers may also only be able to fully evaluate the work if they possess a comprehensive knowledge of the original research included. Together, these issues present a challenge for ensuring quality and rigour in publications.

## Closing points

This study was conceived by the editor-in-chief of *BJPsych Open* (K.R.K.) in response to concerns about the quality of systematic reviews in this and other reputable journals, specifically in relation to transparency and bias minimisation. This study is considered important and timely in light of recent reports highlighting a low prioritisation of reporting transparency and questionable research practices by indexed scientific journals,^90^ which has led to a frequent occurrence of errors. This issue, combined with the rapid growth in systematic review publications,^91^ has significant implications, as biased conclusions can profoundly impact research, clinical and public health domains. Given the importance of bias minimisation in systematic reviews, adhering to best practice guidelines is important. It is also resource-intensive and we therefore urge review authors to plan reviews such that they are both informative and feasible within these parameters. This is a point for consideration even in generating a research question/objective and review scope. For instance, very large systematic reviews carry practical challenges but have the benefit of pooling substantial evidence, which increases their potential impact (and by extension the potential harm of biased findings). We finally emphasise that the foundation of a minimally biased systematic review is in consistently maintaining transparency, thoroughness and reproducibility.^92^

## Data Availability

Data sharing is not applicable to this article as no new data were created or analysed in the course of this study.
